# Physiotherapists transgressions lodged at the Health Professions Council of South Africa between 2010 and 2020

**DOI:** 10.4102/sajp.v80i1.2090

**Published:** 2024-11-29

**Authors:** Nokuzola D. Dantile, Nombeko Mshunqane, Cornelius W. van Staden

**Affiliations:** 1 Department of Physiotherapy, Faculty of Health Sciences, University of Pretoria, Pretoria, South Africa; 2 Centre for Ethics and Philosophy of Health Sciences, Faculty of Health Sciences, University of Pretoria, Pretoria, South Africa

**Keywords:** ethics, transgressions, physiotherapists, unprofessional conduct, HPCSA, medical aid scheme

## Abstract

**Background:**

Complaints of ethical and professional misconduct are lodged and processed by the Health Professions Council of South Africa (HPCSA) in accordance with their legal mandate.

**Objectives:**

This study describes the nature and frequency of transgressions by physiotherapists as concluded by the HPCSA for the period from 2010 to 2020.

**Method:**

A total sampling method was used to extract all records of transgressions lodged against physiotherapists between 2010 and 2020. In a quantitative retrospective records review design, data were captured with the objective to report these descriptively. Ethics approval was granted by the Faculty of Health Sciences Research Ethics Committee of the University of Pretoria and permission to use the records was granted by HPCSA.

**Results:**

Twenty-one transgressions by physiotherapists during the study period were recorded at the HPCSA. Most transgressions were charging for services not rendered (*n* = 20 times), invoices drafted inaccurately (*n* = 17) and false claims submitted to the medical aid schemes (*n* = 15). Other transgressions included failure to obtain informed consent and patient identity prior to treatment, charging for an unkept appointment, overservicing, misleading advertisements, love relationship with a patient and treating an animal in the same practice as humans.

**Conclusion:**

The transgressions were unprofessional in nature with the most frequently reported being false claims and accounts submitted to the medical aid by physiotherapists for services not rendered.

**Clinical Implications:**

The knowledge of transgressions will influence decision making and restrain infringement to enhance sound ethical practice.

## Introduction

The Health Professions Council of South Africa (HPCSA) is a statutory body established by the *Health Professions Act 56 of 1974*, Section 2(1), which functions to determine the strategic policy and make decisions with regard to the professional boards and the health professions, in matters such as finance, education, training, registration, ethics and professional conduct, disciplinary procedure, scope of the professions interprofessional matters and maintenance of professional competence (Section 3[c]) (South African Government 1974). There are 12 professional boards under the ambit of the HPCSA that are responsible for the promotion of the standards of education and training and to guide the professionals on professional practice (*Health Professions Act 56 of 1974*, Section 15A [d], [e] and [h]). Therefore, the Physiotherapy Podiatry and Biokinetics Board sets minimum standards against the scope of the profession of physiotherapy as outlined in the *Health Professions Act 56 of 1974* and is responsible for the maintenance of professional standards through the accreditation of training programmes and compliance thereof (HPCSA 2019). The prerequisite to practise as a physiotherapist is to be registered with the HPCSA (Section 17[1][a] and [2]), and the professional boards have been empowered by the act to institute an inquiry into any complaint, charge or allegation of unprofessional conduct against any person registered under the act and on finding such a person guilty (Section 42[1]) (South African Government 1974).

The physiotherapy profession has experienced an increase in professional autonomy, within the last four decades, thereby increasing the need for formal ethical considerations and self-regulation boundaries (Cooper & Jenkins 2008) that serve to focus more clearly on the individual physiotherapist’s ethical competence: the ability to identify, to examine, to assess and to decide about the ethical issues in daily practice (Richardson 2015). The South African Society of Physiotherapy (SASP) believes that physiotherapists should always act in the best interest of their patients and maintain the highest standards of personal conduct and integrity (SASP 2017). In this regard, Hoffmann and Nortjé (2015) argue that the professional ethics awareness should involve more than mere awareness and/or adherence to HPCSA and/or the SASP codes of conduct.

In South Africa, service providers (physiotherapists included) in private practice are paid by medical schemes on a fee-for-service basis, and members face large co-payments (Ataguba & McIntyre 2012). Australian private healthcare also uses a similar system of fee-for-service private healthcare and has cited instances of billing for unnecessary or unsanctioned services (Hersch et al. 2020). In South Africa and Australia, private practitioners use electronic claims processing systems to the medical aid schemes that are the funders of the private healthcare sector to reimburse the service providers for services rendered to their members (Broomberg & Price 1990). This medical aid re-imbursement system is carried out by the administrators outsourced by the medical aid companies to manage the logistics of processing member claims to pay service providers for services rendered (Department of Health 2005). The prices charged by private practitioners for consultations and procedures reflect their earnings expectations (Ataguba & McIntyre 2012) such that there is a variation in charges to patients. This fee-for-service payment system creates ethical issues for service providers at the point of care further compromising the ethics of physiotherapists as evidenced in the study by Hoffmann and Nortjé (2015) who found that a large percentage (70.3%) of transgressions committed by physiotherapists involves fraud. Legotlo and Mutezo (2018) concurred with the findings that the most reported fraud committed by service providers was the submission of false claims and claims for services that were not rendered to the medical aids. Fraud committed in private health insurance services in Australia was judged as the most expensive (Hersch et al. 2020). A study carried out in Singapore reported clinical ethics issues physiotherapists are faced with because of being both a clinician and a businessman, as this led to maximising profits through overcharging, overservicing and maximising insurance claims (Lim, Xafis & Delany 2023).

Being a good healthcare practitioner requires a life-long commitment to sound professional and ethical practice, making the practice in the healthcare profession a moral enterprise (HPCSA Ethical rules 27A 2022). Despite the moral, ethical and sound ethical practices expected from registered physiotherapists, transgressive behaviour occurs against vulnerable patients (Hoffmann & Nortjé 2015) who rely on the healthcare providers not to abuse their trust (Gerritse & Duvivier 2021). According to the *Health Professions Act 56 of 1974* Section 42(1), any registered health practitioner found guilty of improper or disgraceful conduct after a determination made by a preliminary committee of inquiry or an inquiry held by a professional conduct committee shall be liable for a penalty (South African Government 1974). The study carried out by Hoffmann and Nortjé (2015) reported notable penalties including fines of R5000.00 for improper professional role and ethical misconduct of HPCSA registered physiotherapists on the charges of guilt. A similar system where practitioners may be subjected to disciplinary tribunals exists in the Netherlands, and all hospitals and healthcare practices are obliged to have an internal system for patients to file complaints (Gerritse & Duvivier 2021).

The other transgressions as reported by Hoffmann and Nortjé (2015) included negligence in evaluating, treating or caring for patients and negligence regarding patient documents or records. The improper professional conduct included romantic relationships with patients who are their clients and negligence in caring for patients. Pezdek and Dobrowolsk (2023) argued that a caring physiotherapist should be aware of the extra therapeutic meaning of touch and respect the emotional, psychic and physical boundaries of a patient and/or client while he or she does their job. The issuing of misleading, inaccurate and/or false medical statements together with false and/or inaccurate medical aid claims involving non-rendered services and failure to timeously submit account statements to the relevant medical aid scheme was found to be the most important specific ethics misconduct linked to fraudulent conduct (Hoffmann & Nortjé 2015). Professional ethics awareness should involve more than mere awareness and/or adherence to HPCSA and/or the SASP codes of conduct (Hoffmann & Nortjé 2015). The primary purpose of penalties for ethical transgressions is to protect the public and healthcare professional standards, in terms of providing a deterrent to others (Section 41[1]) (South African Government 1974). This paper describes the nature of transgressions lodged against physiotherapists who violated the ethics, the patient professional relationship or trust and have been reported at the HPCSA and thus have been found guilty of professional misconduct.

## Research methods and design

A quantitative cross-sectional retrospective record review study design was used (Pefile, Mothabeng & Naidoo 2019). Records of registered physiotherapists who were found guilty of professional, ethical or medical-related transgressions from 2010 to 2020 were included. All records that were legal in nature were excluded. Ethical clearance, reference number 508/2021, was approved by the research ethics committee from the University of Pretoria. Permission to peruse the records at the HPCSA was granted by the Registrar of the HPCSA prior to the commencement of the study. A total sampling method was used to select records with transgressions lodged against registered physiotherapists. A pilot study was conducted using a data-collection sheet that included information such as the Physiotherapy registration number, the number of years the physiotherapists have been in practice, area of practice whether hospital or practice rooms, if the physiotherapist appeared at the inquiry, gender, number of charges, types of offences, penalty imposed and nature of complaints. During the pilot phase, it was evident that the HPCSA records were void of some of the information on the data-collection sheet. On careful consideration of the objectives of the study, information such as the number of years in practice, area of practice, appearance at the inquiry and penalty imposed was eliminated although some of it would have added value to the study. A pre-piloted and modified data-collection sheet was used to gather information about the transgression against the physiotherapist, the nature of transgressions (e.g. professional misconduct or fraud) and who the type of complainant was (whether it was a patient, medical scheme or from the physiotherapist). The sourced documents contained finalised cases of all professionals registered with the HPCSA for the period 2010–2020. The physiotherapists were identified by the HPCSA PT registration number and the gender using the tittle of Mr, Miss or Ms, and there were no tittles of Dr used. The identities of physiotherapists were protected by using codes during data capturing. Information extracted included the nature of the transgression, gender and number of counts for the transgression and the entity or person who reported the physiotherapist. The reported transgressions were double-checked by going through the documents at least three times by the first author, through identifying the HPCSA PT numbers for physiotherapy. Data were analysed using descriptive statistics.

### Ethical considerations

Ethical clearance for the study protocol and to conduct the study, was obtained from the University of Pretoria, Faculty of Health Sciences Research Ethics Committee which approved the research proposal. The ethical clearance was obtained in September 2021 (reference no.: 508/2021). Permission to access records of the finalised cases of physiotherapists charged for misconducts was granted by the HPCSA. The identities of physiotherapists were protected by using codes during data capturing. No names are used in the discussion of our study findings to maintain anonymity.

## Results

There were a total of 875 cases for all health professionals registered at the HPCSA reported between 2010 and 2020 for the period under investigation for the study, but there were 21 physiotherapists reported for transgressions, including a group practice (see Table 1).

**TABLE 1:** The transgressions lodged at the Health Professions Council of South Africa against registered physiotherapists between 2010 and 2020.

Category	Year of report											Total
	2010	2011	2012	2013	2014	2015	2016	2017	2018	2019	2020	
Individual physiotherapists reported	3	0	2	6	1	4	3	2	0	0	1	21
Group practice	0	0	0	1	0	0	0	0	0	0	0	-
Number of charges	5	0	2	12	3	4	12	2	0	0	1	41

The gender was identified by the title (Mr and Mrs or Ms) and the first name(s) of the practitioners, which yielded 10 females and 10 males; the last one was a group practice that could not be identified by gender.

There were 21 transgression records of the finalised physiotherapy cases at the HPCSA from 2010 to 2020. There were no cases reported for the years 2011, 2018 and 2019. The highest number of reported cases was in 2013, followed by 2015, 2010 and 2016; 2012 and 2017 had *n* = 2, and 2020 had *n* = 1.

### The nature and frequency of transgressions

The nature of transgressions reported against physiotherapists was unprofessional conduct, where physiotherapists were charged for claiming and charging for services not rendered, accounts drafted inaccurately, false claims submitted to the medical aid scheme, account statements not submitted timeously to the medical aid scheme, overcharging, overservicing and fraudulently charging an ICD10 code not related to the treatment rendered (Table 2). The core professional transgressions included failure to obtain informed consent, charging a patient for an unkept appointment, account statement not being submitted timeously to the medical aid scheme, failure to establish the true identity of the patient before treatment, having a love relationship with a patient, fellow practitioner brought to disrepute on social media, misleading advertisement on a letterhead, advertised services in an untruthful manner, advertised social media franchise, touted and or canvassed for patients failure to attend HPCSA consultations and exposing a patient to danger by treating an animal in the same practice with humans.

**TABLE 2:** The nature and frequency of transgressions (*N* = 71).

Transgressions	Transgression cases	
	Frequency	%
**Nature of transgressions**		
Charging for services not rendered	20	28.17
Accounts drafted inaccurately	17	23.94
False claims submitted to the medical aid scheme	15	21.12
Account statement not submitted timeously to the medical aid scheme	1	1.41
Overcharging, claimed for a minor child as treated over 2 days instead of 1.5 h	1	1.41
Overservicing, failed to apply rule 001 modifier as per NHRPL for failure to keep an appointment†	1	1.41
Fraudulently charging an ICD 10 code not related to treatment	1	1.41
**Perform treatment/intervention without obtaining patient consent**		
Failure to obtain informed consent	2	2.81
Charging for unkept appointment	1	1.41
**Improper professional conduct**	1	1.41
Failed and/or neglected to respond to the Council regarding attending the consultation	1	1.41
Failure to establish the true identity of the medical aid holder before treatment, resulting in a fraudulent claim	1	1.41
Having a love relationship with a patient	1	1.41
Fellow practitioner brought to disrepute on social media	1	1.41
Performed neck manipulation not informing the patient of possible risks and complications	1	1.41
Misleading advertisement on a letterhead – ‘Healthcare’	1	1.41
Advertised social media franchise contract for sale	1	1.41
Advertised services in an untruthful manner	1	1.41
Canvassed or touted or allowed canvassing or touting to be done for patients on your behalf	1	1.41
Exposing a patient to danger by treating an animal (dog) in the same practice with humans	1	1.41

**Total**	**71**	**100.00**

Note: The ICD 10 Code is the global standard for classifying and coding mortality and morbidity data. The NHRPL was a list of tariffs for health services and procedures published by the South African Department of Health.

ICD 10, International Classification of Diseases Tenth revision; NHRPL, National Health Reference Price List.

† , 001 modifiers = timeous cancellation of an appointment, relevant consultation payable.

The number of count of charges per physiotherapist ranged from 1 to 12 charges per physiotherapists. The number of counts for other non-physiotherapy practitioners went up to 33, but there was only one physiotherapist who had 12 counts.

In this study, most complaints were filed by the medical aid followed by patients and lastly fellow practitioners.

## Discussion

This study describes the transgressions committed by the registered physiotherapists at the HPCSA. Our retrospective record review yielded a total of 21 physiotherapists who were found to have ethical transgressions across the study period of 10 years reported at the HPCSA between 2010 and 2020. There seems to be a decline in ethical transgressions in this study compared to the previous study conducted by Hoffmann and Nortjé (2015) where there were a total of 37 transgressions across the study period (2007–2013) for an annual average of 5709 registered physiotherapists, whereas there were 8058 in 2020. The noted decline in ethical transgressions, according to the reported statistics in the HPCSA 2019/2020 Annual report, was the backlog in processing complaints during the reporting phase, whereby 1239 complaints were received during the review period for all the boards, and only 513 matters were finalised. From the 21 cases reported for the study period, there was an equal number of males and females, *n* = 10, with a group practice whose gender could not be ascertained. The equal gender representativity findings are not to be expected considering the findings of the study by Louw et al. (2020) who described the demographic patterns of the HPCSA registered physiotherapists from 1938 to 2018 to be female-dominated professions at 82.9%. Feldman et al. (2023) concur with these findings whereby 73% of physiotherapists are women in Canada and 76% in Quebec. However, in this study, there is no significance of gender as both genders equally transgressed professional ethics.

### Charging for services not rendered and charging for unkept appointments

The maximum transgression found in the study was charging for services not rendered, which constituted 28.17% of the complaints against physiotherapists who were fraudulent in nature. Health practitioners are prohibited from charging for services not personally rendered in terms of the HPCSA Ethical Rule 7, 2022. The study by Hofmann and Nortjé (2015) reported similar transgression results of the high incidence of fraudulent conduct, which accounted for 70.3% during the study period (2007–2013). Similar results of physiotherapists found guilty of malpractice were reported by a study carried out in Canada, which reported 82 decisions by the PPQ disciplinary tribunal between January 2015 and July 2020 (Feldman et al. 2023). Fraud was defined by Graziella, Viorel and Ştefan (2011) as an act of bad faith usually committed by someone to realise a material profit because of breaching another person’s rights, which is evidenced by the physiotherapists found guilty in this study and further described fraud to simultaneously being a crime and a violation of civil rights. Although fraud and abuse are typically viewed by policymakers as financial problems, fraud and abuse perpetrators engage in several activities that could also harm patient health (Hersch et al. 2020), which is beyond the scope of this study. Physiotherapists need to be aware of the importance of balancing the financial and clinical aspects of the business because ethical business practice is at the core of a successful business and can contribute to greater public trust and sustainability (Lim et al. 2023).

Charging for an unkept appointment constituted 1.41% of the transgressions. According to ethical guidelines of the HPCSA Ethical Rule 7 (2022), an appointment that was not honoured by the patient is equivalent to services not rendered, and for that, a practitioner may not charge or receive fees. However, the motivating factor to charge for unkept appointments in private practice is that the physiotherapists are reimbursed (partly) by fee-for-service or per case by the medical aid schemes, and income is lost in case of a no-show appointment, particularly if it cannot be replaced by another patient (Bech, 2005). Notwithstanding the loss of income, charging for an unkept appointment is transgressing the ethical rules (HPCSA Ethical Rule 7 2022), which states that ‘A practitioner shall not charge or receive fees for services not rendered’. Delany (2007) and Lim et al. (2023) are of the opinion that physiotherapists are to observe clinical and professional boundaries and ethical issues related to business relationships and operations, thus avoiding ethical transgressions.

### Inaccurately drafted accounts and false claims

The accounts drafted inaccurately accounted for 23.94% of the fraudulent transgressions. Similar results of malpractice regarding fraudulent documentation and billing of services were reported by Feldman et al. (2023) in Canada. Legotlo and Mutezo (2018) concurred that the service provider fraud most reported by the participants in their study was the false claims submitted to the medical scheme, even though the services were not rendered or products not supplied to the members. A study conducted in Romania by Graziella et al. (2011) reported similar findings that physiotherapists were claiming for services not performed or claiming more services than supplied and highlighted financial pressure as the incentive that motivated the fraudulent behaviour. The fraudulent behaviour by physiotherapists in dealing with the billing of services seems to be widespread. In this study, the false claims submitted to the medical aid accounted for 21.12% of the nature of transgressions, which is particularly concerning considering the HPCSA ethical guidelines on non-maleficence, which state that healthcare practitioners must not harm or act against the best interests of patients, even when the interests of the latter conflict with their self-interest (HPCSA Ethical Rule 27A 2022). To prevent future misconducts, colleagues must be encouraged to be whistleblowers by reporting professional misconduct so as to potentially promote professionalism and curb the ethical issues of fraudulent behaviour that is being faced in the profession (Mansbach, Melzer & Bachner 2012).

### Account statement not submitted timeously to medical aid scheme

There was a physiotherapist who was charged for an account statement that was not submitted timeously to the medical aid scheme. It is a requirement of the *Medical Schemes Act 131 of 1998* and the rules of the fund that claims be submitted no later than the last day of the fourth month after the last day of service was rendered (South African Government 1998). Therefore, any claims submitted after this period will not qualify for payment by the medical aid scheme. The complaint against the physiotherapist was valid as the claim was submitted 4 months after the last date of service. There is dearth of literature on this transgression; however, this points out to lack of business administration skills if a physiotherapist fails to process and submit a claim to the medical aid scheme for a period of 4 months. The first UK-based study exploring the business skills and experiences of private physiotherapy clinic owners found the participants confident in discussing clinical policies, but five out of six participants felt that additional physiotherapy-related business training would be beneficial (Watson & Lowe 2023). In this regard, support and guidance in the physiotherapy-related business including the administration would be beneficial for the physiotherapist who fails to submit claims timeously.

### Charging an ICD 10 code not related to treatment

There was a physiotherapist charged for fraudulently charging an ICD 10 code not related to the treatment rendered. Findings similar to our study were reported by Legotlo and Mutezo (2018) regarding irregular billing of codes, whereby service providers claimed for a code of a higher value than the actual treatment provided, thus manipulating codes by billing for extra codes and billing for several codes instead of one inclusive code to defraud medical schemes. It needs to be argued that the fee-for-service reimbursement system used by the medical aids in South Africa contributes to the financial manipulation of the ICD 10 code for greater financial rewards as observed in the study by Cantu (2019) who reported that the prospective payment system drives reimbursement. It would seem that the purpose of an ICD 10 code not related to treatment was to defraud the medical aid; hence it is a transgression. Therefore, the physiotherapists failed to adhere to the ethical principles as set out in HPCSA Ethical Rule 17 (2022) regarding the ICD 10, which states that there must be a full and frank disclosure of the treatment undertaken.

### Overservicing, overcharging

There were a low number (1.41%) of physiotherapists who were charged for both overcharging and overservicing. The data provided did not indicate the year of qualification that would indicate the number of years in practice to align with findings by Fryer et al. (2021) that working longer years in physiotherapy and learning about ethics in basic Physiotherapy education was associated with participants reporting lower frequencies of ethical issues. However, there is a low percentage of overservicing and overcharging; it is a transgression that is prohibited by the HPCSA ethical guidelines because there is improper financial gain of the physiotherapist that is contrary to ethical or professional rules (HPCSA Ethical Rule 7 2022). A similar study carried out in Singapore among private practice physiotherapists reported clinical ethical issues faced by physiotherapists that led to maximising profits through overcharging and overservicing (Lim et al. 2023), indicative of the widespread prevalence of ethical challenges faced by private practitioners.

### Failure to obtain informed consent

In the study, the failure to obtain informed consent accounted for 9.52% of the transgressions. This finding could be associated with a lack of training in ethics, which is similar to the study by Fryer et al. (2021) who associated the high frequency of ethical issues with the importance of developing a strong ethics curriculum in the training of 21st century physiotherapy graduates. Copnell (2018) stated that physiotherapists have a professional and moral duty to enable patients to make good decisions about the treatment they are to receive through the informed consent, which is at the centre of the patient–therapist relationship. A study carried out by Aderibigbe and Chima (2019) on physiotherapists and assistants in KwaZulu-Natal public healthcare institutions demonstrated insufficient knowledge by physiotherapists and assistants on informed consent. The lack of knowledge is concerning as the HPCSA ethical guidelines stipulate that practitioners should always seek informed consent from patients ahead of providing any treatment (HPCSA Ethical Rule 27A 2022). The successful professional relationships between healthcare practitioners and patients in private practice need to focus on ethical issues surrounding informed consent and power asymmetry (Delany 2007). Aderibigbe and Chima (2019) therefore recommended that regular updates on ethics and healthcare law help to bridge the knowledge gap.

### Having a love relationship with a patient

There was a physiotherapist who was charged with having a love relationship with a patient. Similar findings were reported in a study conducted in Canada and Quebec, where two women and one man were found guilty of having had an intimate relationship with a patient (Feldman et al. 2023). Professional boundaries are the parameters that dictate the expected behaviour between a health professional and the patient within the therapeutic relationship (Cooper & Jenkins 2008), and physiotherapists need to be aware of the limits of such boundaries as they may be at higher risk of boundary violations. Gerritse and Duvivier (2021) explained these boundary violations to include having a sexual relationship that causes physical, mental or emotional damage to patients. In New Zealand, the code of ethics adopts a zero-tolerance stance where practitioners violate sexual boundaries with patients (Surgenor, Kate & Marta 2019). The study by Feldman et al. (2023) raised an interesting finding on sexual misconduct that there were more complaints for men, with nine men having committed sexually abusive actions (12 guilty disciplinary decisions), while no women were found guilty of sexual abuse of patients. However, this finding will require more research into the gender difference in committing sexual misconducts.

### Disrepute on social media

There was a fellow practitioner who was brought to disrepute on social media, which is unethical because the HPCSA ethical guidelines clearly state that practitioners should refrain from speaking ill of colleagues or other healthcare practitioners (HPCSA Ethical Rule 12 2022). The use of social media has increased exponentially throughout the world to provide a platform for building social and professional relationships that can be used by all, including healthcare professionals who ought to ask themselves before posting on social media whether sharing certain information is legally and morally defensible, or it reflects the professional conduct expected of them (Kubheka 2017). The social media includes social networks (e.g. Facebook, Twitter, WhatsApp and LinkedIn), content-sharing platforms (e.g. YouTube and Instagram), personal and professional blogs (including email, SMS, electronic journals as well as those published anonymously), internet discussion forums and the comment sections of websites (HPCSA Ethical Rule 9 2022). Thus, all these social media platforms are subject to ethical scrutiny. Although the health practitioners may engage fully in debates on health matters, they must be aware that the laws regarding defamation, hate speech and copyright also extend to the content shared via social media (HPCSA Ethical Rule 12 2022).

### Misleading advertisement on social media and touting of patients

There were transgressions of misleading advertisement on a letterhead, which advertised services in an untruthful manner and advertisement of a social media franchise. The HPCSA allows practitioners to advertise their services but prohibits advertisement that is unprofessional, untruthful, deceptive or misleading or causes consumers unwarranted anxiety (HPCSA Ethical Rule 3 2022). Simpson (2019) reiterates that consumers need to be provided with ethical accurate advertising to aid with making informed health-related decisions and further states that unacceptable advertising has the potential to cause harm. There is a physiotherapist who was found to have touted for patients and thus transgressed the ethical rule that states that ‘a practitioner shall not canvass or tout or allow canvassing or touting to be done for patients on his or her behalf’ (HPCSA Ethical Rule 3 2022). Simpson (2019) concurs that all practitioners must abide by the advertising guidelines and act in the best interests of patients. To circumvent these unethical behaviours, Cantu (2019) suggests that the physical therapy students need to be made aware of moral responsibilities and to understand the contextual intricacies of managing a healthcare business.

### Identity of the medical aid holder before treatment and failure to respond when summoned by Health Professions Council of South Africa

The physiotherapist who failed to establish the true identity of the medical aid holder did not comply with ethical standards because it is compulsory to identify the personal elements of a patient (HPCSA Ethical Rule 15 2022). Phipps et al. (2012) concur with the importance to identify the individual as the person for whom the treatment is intended as misidentification may occur at any time throughout the course of a patient’s treatment. There was a physiotherapist who failed to respond when summoned to appear at the disciplinary hearing at the HPCSA, transgressing the HPCSA ethical guidelines, to comply with any lawful instruction, to attend a consultation at the time and place stipulated by the council or official of council (HPCSA Ethical Rule 27A 2022). Ogunbanjo and Van Bogaert (2014) alluded to the continued presence of a small percentage of individuals who will opt for misconduct while being fully aware of the difference between ethical conduct and misconduct.

### Limitations

The records of transgression from the HPCSA did not indicate the number of years each individual physiotherapist has been in practice, which could have aided in establishing the relationship of working for longer with less frequent occurrence of ethical issues in physiotherapy practice. Further research to explore factors that influence the ethical practice among physiotherapists is needed as this will assist to determine the type of ethics topics to be included in the undergraduate physiotherapy curriculum.

### Recommendations

To improve conduct, physiotherapy-related business training needs to be included in the undergraduate curriculum that will include training regarding taking payments and reimbursement systems utilised in private practice. Advocacy programmes will also need to be initiated by the professional society of physiotherapy to look into reimbursement models and systems that will reduce pressure on physiotherapists in private practice, to get fair payment from the medical aids for services rendered. Further research will need to be undertaken to look into the effectiveness of penalties imposed to aim to deter reoffending.

## Conclusion

There were 21 physiotherapists who were found guilty of ethical transgressions at the HPCSA between 2010 and 2020. The most frequent committed transgression found in the study was the charging for services not rendered, followed by inaccurately drafted and false claims. These transgressions are indicative of the need to create strategies that include physiotherapy-related business support and guidance to curb ethical misconducts and transgressions as well as to rehabilitate physiotherapists into ethical professional practices. The other transgressions included unprofessional conduct, claiming from the medical aid scheme for services not rendered, accounts drafted inaccurately, submission of false claims, account statements not submitted timeously to the medical aid scheme, overcharging, overservicing and fraudulently charging an ICD10 code not related to treatment. The core professional complaints included failure to obtain informed consent, charging a patient for an unkept appointment, failure to establish the true identity of the patient before treatment, having a love relationship with a patient, fellow practitioner brought to disrepute on social media, misleading advertisement on a letterhead, services advertised in an untruthful manner, advertised social media franchise, touted and or canvassed for patients failure to attend HPCSA consultations and exposing a patient to danger by treating an animal in the same practice with humans.

Given the frequency at which ethical problems are reported, solutions and strategies to resolve these types of transgressions point to the importance of the need for the universities to develop a strong undergraduate ethics curriculum in the training of 21st-century physiotherapy graduates.
